# An aggressive form of non-Hodgkin's lymphoma with pleural and abdominal chylous effusions: A case report and review of the literature

**DOI:** 10.3892/ol.2013.1501

**Published:** 2013-07-29

**Authors:** YAJIAN JIANG, WANZHUO XIE, KEYUE HU, JIE SUN, XIAOLI ZHU, HE HUANG

**Affiliations:** 1 Department of Hematology, Bone Marrow Transplant Center, The First Affiliated Hospital of Zhejiang University School of Medicine, Hangzhou, Zhejiang 310003, P.R. China; 2 Program of Clinical Medicine, School of Medicine, Zhejiang University, Hangzhou, Zhejiang 310003, P.R. China

**Keywords:** non-Hodgkin's lymphoma, pleural effusion, ascites

## Abstract

Serous effusions, including pleural, abdominal and pericardial effusions, are complications of lymphoma. Among these types, pleural effusions are the most common to be observed. However, the involvement of the abdominal or pericardial cavity is rare. An impairment of the lymphatic drainage and direct infiltration have been identified to play significant roles in effusion formation. Multiple techniques, including cytological exams, immunochemistry and cytogenetics, have been applied in the clinic to access the qualities of the effusions and to attain a fast and precise diagnosis. Serous effusions are associated with a poor outcome for patients with lymphoma. The present study describes the case of a 28-year-old male patient with aggressive non-Hodgkin's lymphoma (NHL) involving pleural and abdominal chylous effusions.

## Introduction

Serous effusions are complications that are observed in lymphoma patients (1–4). Statistics show that in patients with non-Hodgkin's lymphoma (NHL) and Hodgkin's disease (HD), 20–30% will develop a pleural effusion (3,5). However, effusions in the peritoneal and pericardial cavities are uncommon (5). Of all the various subtypes, T-cell originated lymphomas, particularly lymphoblastic lymphoma, usually involve serous effusions (5–7,9). The main reasons for pleural effusions in HD patients are an obstruction of the thoracic duct and impaired lymphatic drainage (8). In NHL, the primary mechanism for effusions is direct pleural infiltration (9). Cytological examinations of the effusions may occasionally be of use. The percentage of positive cytological findings may vary widely between NHL pleural effusions (22.2–94.1%) (8). To attain a more precise diagnosis, immunocytochemistry (ICC), morphometry, flow cytometry (FCM) and cytogenetics have been applied in the clinic. Effusions are generally associated with a poor outcome for patients with lymphoma (5,9). The present study describes the case of a 28-year-old male patient with aggressive NHL involving pleural and abdominal chylous effusions. Furthermore, a detailed review of the literature on the pathogenesis of serous effusions in lymphoma and possible treatment plans is discussed. Written informed consent was obtained from the patient.

## Case report

A 28-year-old male patient was admitted to The First Affiliated Hospital of Zhejiang University School of Medicine (Hangzhou, Zhejiang, China) in January 2012 for continuous treatment for NHL. The patient had suffered abdominal pain 5 months prior to admittance, for which he was treated at a local hospital. Abdominal computed tomography (CT) revealed a mass in the bowels and retroperitoneal lymphadenopathy. The patient was sent for abdominal exploratory surgery and the excised mass was identified to be a lymphoma with positive results for leukocyte common antigen (LCA), CD20 and CD79a. The cytogenetic analysis showed a 46,XY,t(8,14)(q24,q32) translocation. The hematologist administered a cycle of DOLP (40 mg/m^2^ daunorubicin on days 1–3; 1.4 mg/m^2^ vincristine on days 1, 8, 15 and 22; 6,000 U/m^2^ L-asparaginase on days 11–20; and 45 mg/m^2^ prednisone on days 1–28) plus CTX (750 mg/m^2^ cyclophosphamide on day 1), and then a cycle of HDAra-C (3 g/m^2^ high-dose cytarabine, every 12 h on days 1–3) plus 6,000U/m^2^ L-asparaginase on days 4–13 and a cycle of VDCP (1.4 mg/m^2^ vincristine on days 1, 8, 15 and 22; 40 mg/m^2^ daunorubicin on days 1–3; 750 mg/m^2^ cyclophosphamide on days 1 and 8; and 45 mg/m^2^ prednisone on days 1–28). The patient was stable following the treatment. Following this, the patient suffered mild abdominal pain and a cough and was admitted to The First Affiliated Hospital of Zhejiang University School of Medicine hospital for further treatment of the lymphoma.

**Figure 1:**
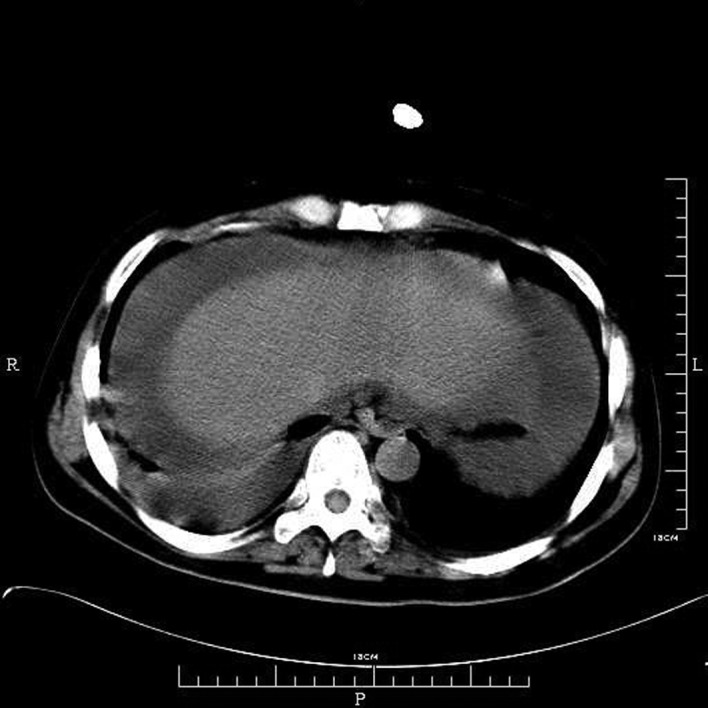
Serous effusions. CT showing the right pleural and abdominal effusions. CT, computed tomography.

Upon admission, the patient experienced mild abdominal pain and a temperature of 38ºC. Small lymph nodes around the neck region could be palpated. The sternal pressing sign was negative. The liver and spleen were not enlarged upon palpation. An abdominal shifting dullness test was positive. The patient produced a white, non-sticky sputum as a result of coughing. Although the patient was able to breathe freely, a small amount of chest distress was observed. The patient was sent for a chest and abdominal CT and the results revealed pleural and abdominal effusions (Fig. 1).

The patient was prescribed antibiotics and a whole body evaluation was performed prior to another round of chemotherapy. However, the patient's condition worsened at a speed greater than expected. Initially, the pleural effusion caused difficulty in breathing, then the patient's temperature increased further. The antibiotic dose was adjusted based on the sputum culture results, and a pleurocentesis and abdominocentesis were performed to remove the pressure. The effusions were milk-colored, indicating chylous effusions. The biochemistry results confirmed that the effusions were chylous (Table I). The cytological studies identified 89.62% aberrant B lymphocytes, of which, 20.93% were CD79a-positive, 35.10% were CD5-positive, 99.50% were HLA-DR-positive, 27.24% were CD10-positive, 88.38% had λ chain expression, 96.58% had κ chain expression, 63.78% were cIgM-positive and 65.80% were sIgM-positive, as determined by FCM.

As the infection was believed to be under control, the patient was administered VMCP chemotherapy (1.4 mg/m^2^ vincristine on days 1, 8, 15 and 22; 10 mg/m^2^ mitoxatrone on days 1–3; 750 mg/m^2^ cyclophosphamide on days 1 and 8; and 45 mg/m^2^ prednisone on days 1–28). At the end of this round of chemotherapy, the patient was again febrile and had a severe cough. The patient eventually succumbed due to septic shock.

## Discussion

Serous effusions occur in a number of malignancies (1). Johnston *et al* studied 584 patients with serous effusions and identified that 15% were due to lymphoma (3). Other causes include various types of cancers, tuberculosis and renal disease (1,4). Among the total lymphoma patients with serous effusions recorded by Johnston *et al*, 72% were males and 29% were females, suggesting a predominance in males (3). Serous effusions also have another preference in the various subtypes of lymphoma. T-cell originated lymphomas are more commonly observed with serous effusions than B-cell originated neoplasms. Pleural effusions have been identified in 26.2% of T-cell lymphomas, while, in contrast, only rare B-cell lymphomas develop pleural effusions (10,11). Among all the NHL subtypes, 41.6% of lymphoblastic lymphomas have been recorded with pleural effusions compared with only 3.8% of the other subtypes (12).

Various causes may lead to serous effusions in lymphoma patients, including impaired lymphatic drainage due to obstruction in the mediastinal lymph nodes or the thoracic duct, venous obstruction, pulmonary infection, radiation therapy or pleural involvement of the tumor (13). The main cause of pleural effusion in HD has been identified as thoracic duct obstruction. However in NHL, the primary consideration was shown to be direct pleural infiltration (4). Chylous effusions are always caused by an obstruction of the lymphatic trunks. In the present case, the pleural and abdominal effusions were identified to be chylous, strongly indicating that the effusions originated in the lymph trunks. Possible reasons for the effusions may be that the metastatic lymphoma cells blocked the lymph tunnels, leading to obstruction and further impairment of these tunnels.

Removal of the fluids from the thorax by thoracocentesis, from the peritoneal cavity by abdominal paracentesis and from the pericardial cavity by pericardiocentesis, are diagnostic methods and treatments to relieve pressure. The fluids that are extracted from the body cavities may be used to study the disease qualities and to look for malignant cells. Das *et al* compared fine-needle aspiration cytology with pleural effusion cytological studies and identified that in 93.7% of the cases, the cytological findings matched the diagnosis (8). Following the development of new techniques and our improved understanding of biomarkers, immunochemistry, FCM and cytogenetics are being widely used to aid in obtaining a fast and precise diagnosis (14,15). In the present study, a thoracocentesis and a paracentesis were performed. An evaluation of the fluids extracted identified the presence of malignant lymphoma cells, indicating that the serous effusions were actually due to the malignancy. Removal of the fluids aided in the relief of the symptoms and improved our understanding of the etiology.

Patients with lymphoma seldom have any symptoms other than serous effusions. There is a type of lymphoma called primary effusion lymphoma (PEL), which only presents with serous effusions, but no detectable solid masses. Numerous molecular markers have been used to diagnose this lymphoma in the early stage (16–18). PEL has been associated with advanced AIDS. Although PEL is considered to have no detectable solid masses, studies have shown the involvement of certain lymph nodes, tongue-based lesions and the secondary bowel (17–20). The mechanism for PEL has not been well characterized. However, studies have shown that an increased level of vascular endothelial growth factor (VEGF) may induce capillary growth in PEL effusions (21). The diagnosis of PEL mainly depends on the clinical presentation, imaging results and cytological analysis.

Serous effusions in lymphoma are generally associated with a poor outcome (22–24). Relieving symptoms and increasing the quality of life of the patient are the primary treatment targets. In high-grade malignant lymphomas, including Burkitt's lymphoma and lymphoblastic lymphoma, serous effusions are commonly observed and associated with a poor outcome (25–27). If the affected patients are treated with chemotherapy, there is a possibility that a condition called acute tumor lysis syndrome may develop. Studying serous effusions may also aid in the detection of tumor lysis syndrome, therefore, allowing the patient to undergo anti-uric treatment.

In summary, the present study described the case of a young male with NHL involving chylous pleural and abdominal effusions. Serous effusions are common in lymphomas, particularly those of a high grade, including Burkitt's and lymphoblastic lymphomas. However, it is rare for a lymphoma patient to have serous effusions in the pleural and abdominal cavities. The serous effusions of the present case were identified to be chylous. The present case further indicates that serous effusions are formed due to the mechanism of lymphatic trunk obstruction, and that the appearance of serous effusions is associated with a poor outcome, particularly in patients with malignant lymphoma.

## Figures and Tables

**Table I:** Biochemistry laboratory test results for the pleural and abdominal effusions.

Biochemistry exams	Pleural effusion	Ascites
Appearance	Milk-like	Milk-like
WBCs, μl	25000	28000
Lymphocytes, μl	5000	4200
Neutrophils, μl	20000	23800
RBCs, μl	1020	200
LDH, U/μl	4051	4730
ADA, U/μl	34	40
Rivatal test	Positive	Positive
Chylous test	Positive	Positive

WBCs, white blood cells; RBCs, red blood cells; LDH, lactate dehydrogenase; ADA, adenosine deaminase.
